# The “floating ulna” injury in adults: a case report, literature review and proposed injury classification

**DOI:** 10.1186/s12891-020-03880-4

**Published:** 2021-01-06

**Authors:** Tian He, Xin Wang, Shui Sun, Lugang Zhou

**Affiliations:** 1 Department of joint surgery, Shandong Provincial Hospital, Cheeloo College of Medicine, Shandong University, 324 Jingwuweiqi Road, Huaiyin, Jinan, Shandong 250021 P. R. China; 2 Department of Orthopedics Surgery, Yantai Yuhuangding Hospital Affiliated to Medical College of Qingdao University, 20 East Yuhuangding Rd, Zhifu District, Yantai, 264400 Shandong P. R. China

**Keywords:** Galeazzi fracture, Elbow joint dislocation, Floating ulna, Injury classification

## Abstract

**Background:**

Ipsilateral Galeazzi fracture with elbow dislocation, namely the “floating ulna” injury, is a rare injury pattern. A few reports have described this type of injury and its treatment.

**Case presentation:**

A 33-year-old female at 38^+^ weeks gestational age presented with Galeazzi fracture and posterolateral elbow dislocation of the left upper extremity. The patient was treated with closed reduction of the elbow, open reduction, and internal fixation of the radial shaft fracture with a dynamic compression plate and K-wire stabilization of the unstable distal radioulnar joint. At the 12-month follow-up, the patient had no pain or signs of instability. Range of motion was 0–135° at the elbow, 70° extension and 80° flexion at the wrist, and 80° supination and 80° pronation at the forearm.

**Conclusion:**

The “floating ulna” injury is a rare and special injury pattern with ipsilateral Galeazzi fracture and elbow dislocation. This type of injury was likely caused by significant amount of deforming force and the unique position of upper limb when the patient fell from a height of 1–2 m in high-energy trauma.

## Background

The Galeazzi fracture dislocation occurs in 6.8% of all adult diaphyseal forearm fractures [1, 2]. The elbow dislocation accounts for 11–28% of all injuries to the elbow [3]. Only a few cases have reported Galeazzi fracture combined with ipsilateral elbow dislocation [4–10]. The present report describes a rare case of the combination of Galeazzi fracture and dislocation of the left elbow. The mechanism, injury factors, injury classification, and treatment are discussed with a review of the literature.

## Case presentation

**Fig. 1:**
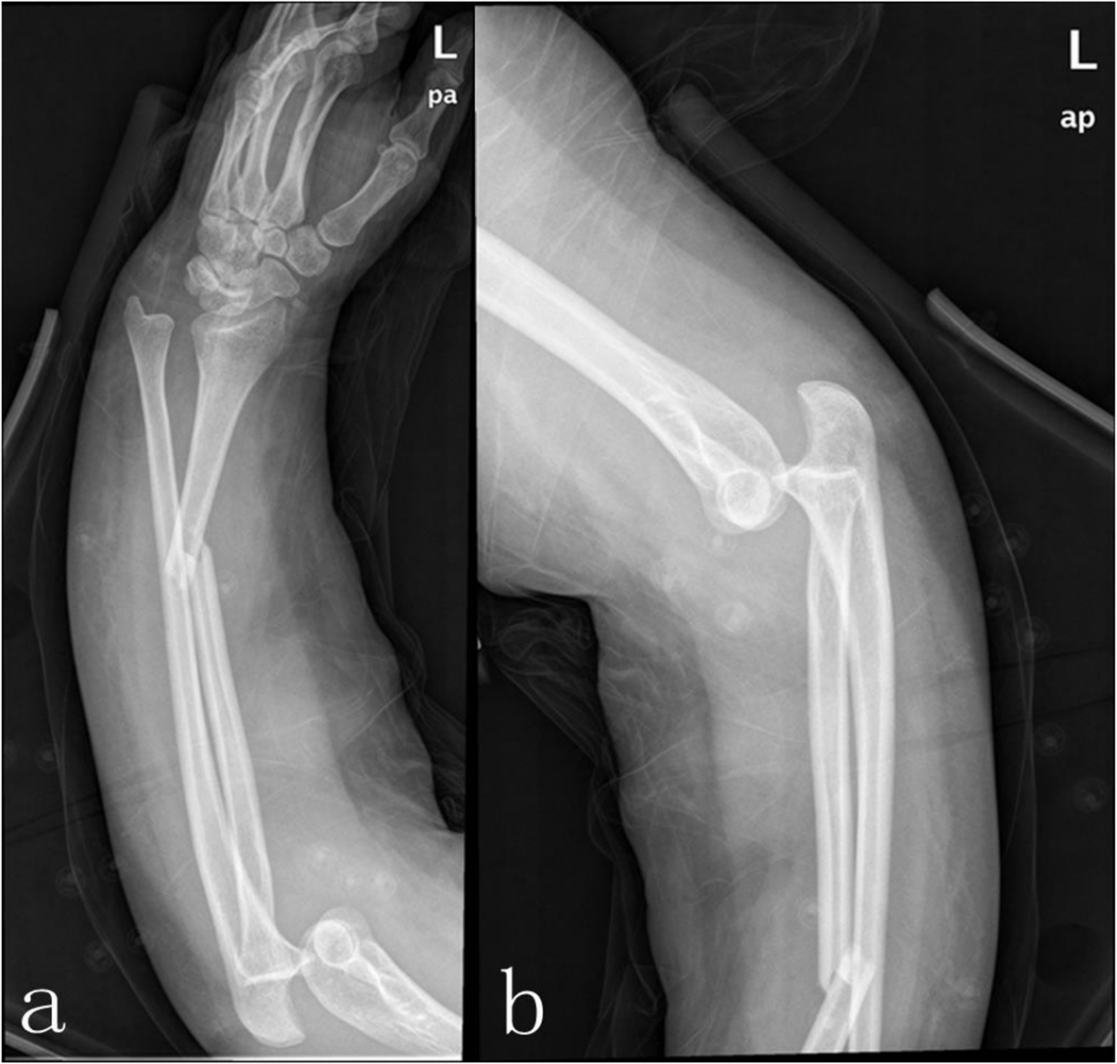
X-ray of the left forearm showing both Galeazzi fracture and elbow dislocation. The fracture of radial shaft at the junction of middle and distal thirds, dorsal dislocation of the ulnar head (a), posterolateral dislocation of the elbow(b)

A 33-year-old female at 38^+^ weeks gestational age presented to the emergency room with left forearm and brain injury, which she sustained after falling off her motorbike in a motorbike accident. She stated that she had fallen on the outstretched left arm. Clinical examination revealed deformity of the elbow and forearm. Bilateral extremities were deformed and swollen obviously. No neurovascular injury was present. Despite a normal prenatal ultrasound and computerized tomography (CT) scan of the brain, X-ray of the forearm showed a transverse fracture of the radial shaft at the junction of the middle and distal thirds, dorsal dislocation of the ulnar head and posterolateral dislocation of the elbow (Fig. 1).

**Fig. 2:**
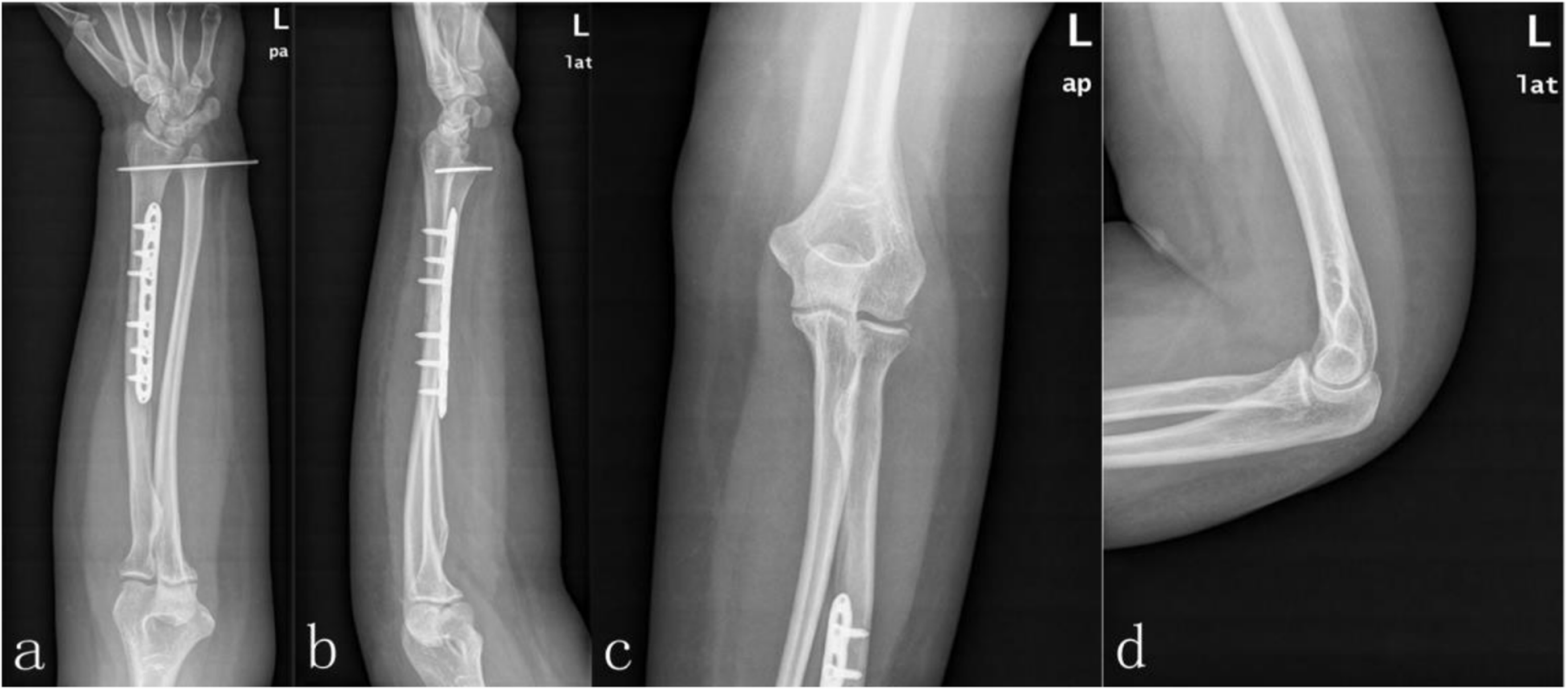
Postoperative radiographs of the left forearm and elbow show successful reduction and fixation of both Galeazzi fracture and elbow dislocation: posteroanterior view of the left forearm (a), lateral view of the left forearm (b), posteroanterior view of the left elbow joint (c), lateral view of the left elbow joint (d)

**Fig. 3:**
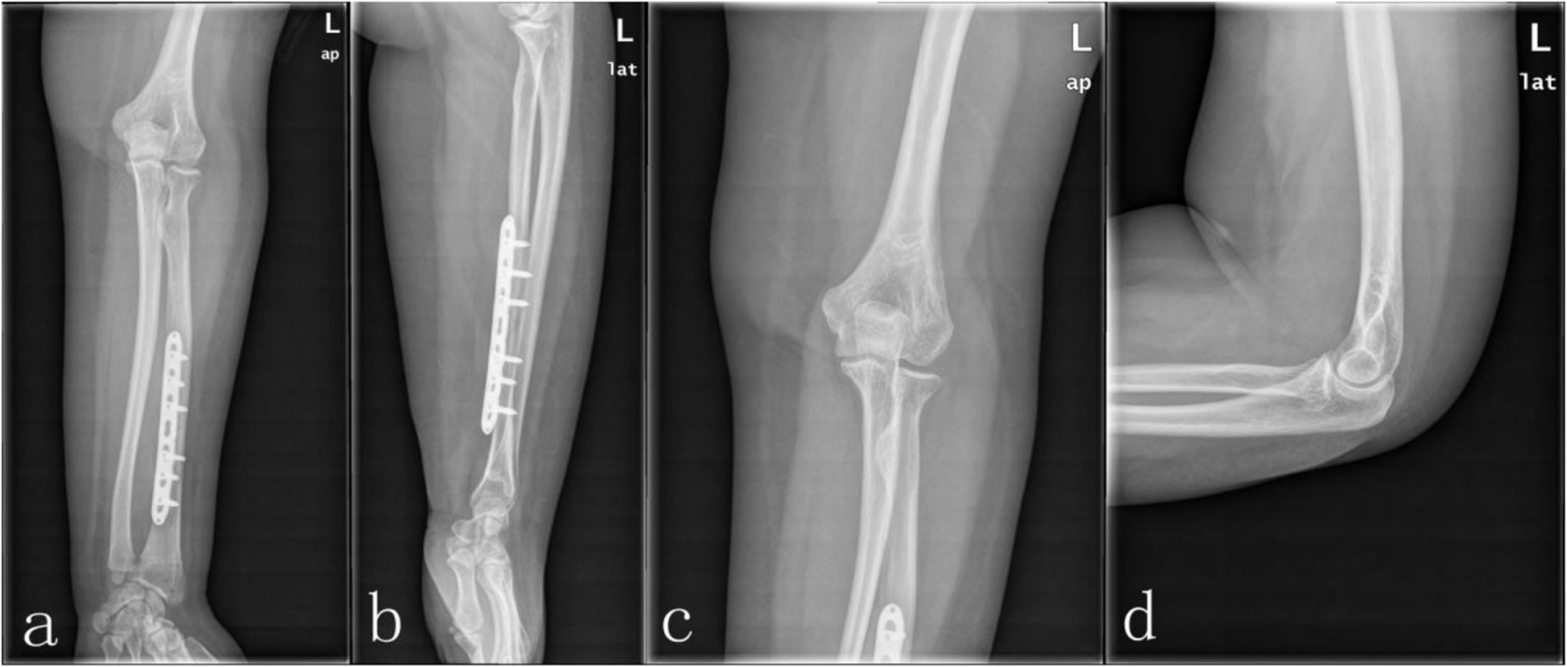
The X-rays of the final follow-up at 12 month

**Fig. 4:**
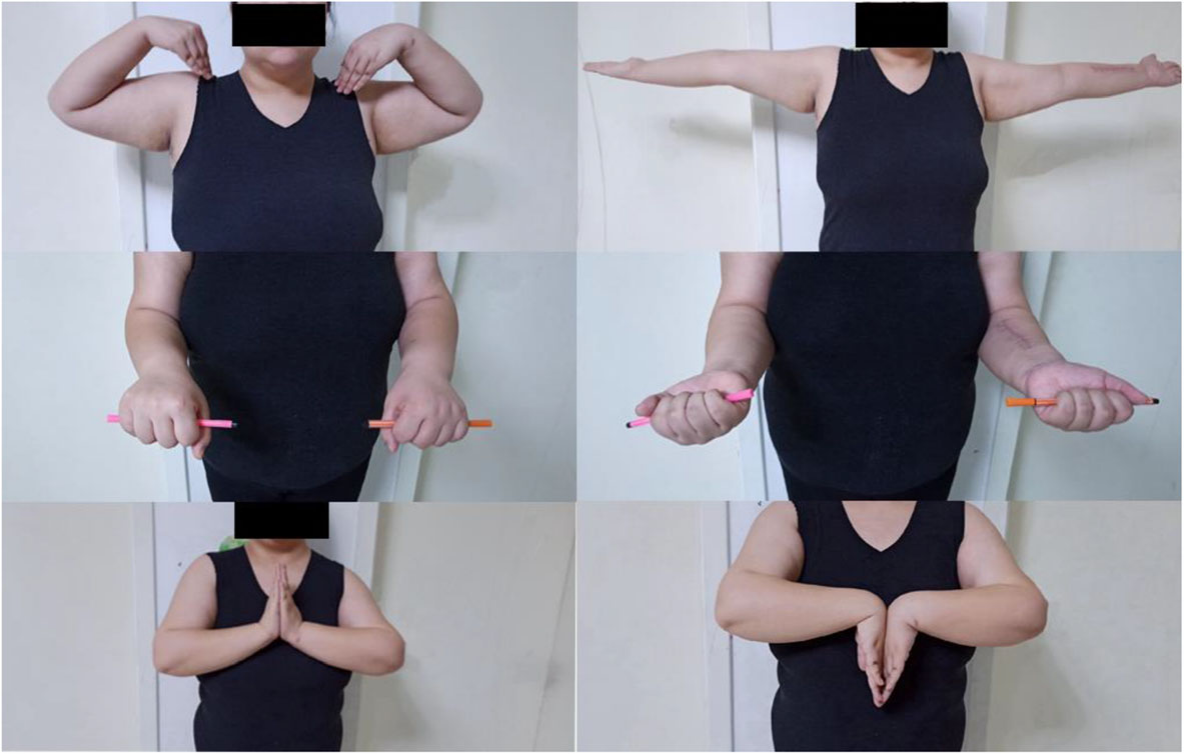
The range of motion at the 12 month follow-up

The patient was taken to the operating room three hours after arrival at the emergency department. Under brachial plexus nerve block, reduction of the elbow dislocation was performed immediately, and radiographs confirmed the elbow to be in joint and tested stability. After that, the patient underwent an open reduction and internal fixation of the radial fracture using a standard palmar approach of Henry [11]. A seven-hole 3.5-mm locking compression plate was used to stabilize the radius. Although the dislocation of the distal radioulnar joint (DRUJ) was anatomically reduced after internal fixation, when the forearm was pronated, dorsal dislocation of the ulna was found under fluoroscopic examination and pinned in neutral position using a 2.0-mm Kirchner wire (K-wire) (Fig. 2). The extremity was immobilized in a long arm plaster slab with the elbow in 90° flexion and the forearm in the neutral position for 4 weeks. Extension exercises of the elbow joint were started with a limited motion brace. After 8 weeks, the K-wire was removed from the DRUJ, and pronation and supination exercises were then commenced. After 12 months, the patient had no pain or clinical evidence of instability (Fig. 3). The range of motion was 0–135° at the elbow, 70° extension and 80° flexion at the wrist, and 80° supination and 80° pronation at the forearm (Fig. 4).

## Discussion and conclusions

Association of the Galeazzi fracture with dislocation of the elbow would result in dislocation of the DRUJ and ulnohumeral joint. The ulna loses the protection of the ligament structure and exhibits a floating condition. This complex injury can be considered a “floating ulna” injury, which differs from the Essex-Lopresti injury, Criss-Cross injury, floating forearm, and floating radius injury of the forearm [12–16].

The mechanism responsible for the “floating ulna” injury in the present case can be considered as due to a transmission of axial forces starting from the outstretched hand combined with extreme pronation of the forearm to full elbow extension. The forceful axial loading from the impact with the ground could have caused the dislocation of the DRUJ, resulting in the radius fractures. Collapse of the radial column would result in transmission of the continuing force through the intact ulna and interosseous membrane of the forearm. The transmitted axial force levers the ulna out of trochlear articulation, causing dislocation of the ulnohumeral joint.

Of the publications, only 7 cases of “floating ulna” injury were strictly screened out [4–10] (Table 1), comprising five male and two female patients. The average age of the 7 patients was 34 years old (26–58 years). According to the accident described in the previous studies (Table 1), falling from a height of 1–2 m was the most likely cause of “floating ulna” injury [4–10]. High-energy trauma in most cases is another cause of the “floating ulna” injury. Nanno et al. [4] reported a case of Galeazzi fractures associated with dislocation of the right elbow and fracture of the right scaphoid caused by a motorbike accident. Ng and Rose [5] described a case caused by car accident combining a Galeazzi fracture, elbow dislocation and fracture of the scaphoid and ulnar styloid. The authors believed that posterior elbow subluxation associated with radial head and coronoid fracture (terrible triad variant) was a result of varus posteromedial rotation force. Sarup and Bryant [6] reported an unusual case of Galeazzi fracture combined with elbow dislocation and an additional ipsilateral humeral shaft fracture resulting from a fall down a flight of stairs. The direction of ulnar dislocation was unclear because the lateral radiograph was not shown. Adanir et al. [7] reported a case that was also a result of a relatively high-energy sports trauma that caused an open Galeazzi fracture and ipsilateral elbow dislocation. The patient in this report likely undergone a motorcycle accident injury with a similar mechanism to that described by Rajeev et al. [8]. However, that case was associated with subacute posterior dislocation of the elbow that was identified five weeks after open reduction internal fixation of the radial shaft fracture. Nevertheless, only one case was caused by a low-energy trauma that sustained a complex injury, with the body weight of the patient acting as a driving force resulting in Galeazzi fracture, elbow dislocation, and radial head fracture [9]. Regarding the Galeazzi fracture in adults, a much greater number of reported ulna-dorsal type than apex- palmar type fractures has been reported [17]. However, according to previous publications (Table 1), the apex- palmar type combined with elbow dislocation occurred in 4 cases, and the apex-palmar type Galeazzi fracture might be more likely than apex-dorsal type to cause “floating ulna” injury. All the patients had successful outcomes, which were achieved by addressing each component of the complex injury individually.

**Table 1:** Previously published studies with “floating ulna” injury in adults

Author	Year	Age/Gender	Mechanism	Injury description			Treatment		
				Type of Galeazzi fracture	Elbow dislocation	Associated injury	Galeazzi fracture	Elbow dislocation	Associated injury
Adanir et al. [7]	2016	26/M	Fall while running	Apex volar type	Posterolateral dislocation	–	Radial plating and DRUJ pinning	Closed reduction	–
Asadollahi et al. [9]	2013	58/F	Fall while running	Apex dorsal type	Posterolateral dislocation	Radial head fracture	Radial plating and DRUJ pinning	Closed reduction	Conservative treatment
Rajeev et al. [8]	2011	26/M	Motorcycle accident	Apex dorsal type	Subacute Posterior dislocation	–	Radial plating	Open reduction	–
Nanno et al. [4]	2011	32/M	Motorcycle accident	Apex volar type	Posterior dislocation	Scaphoid and ulnar styloid fracture	Radial plating and tensionband of ulna fracture	Closed reduction	Herbert screw internal fixation
Ng and Rose [5]	2010	27/M	Car accident	Apex volar type	Posteromedial dislocation	Radial head and coronoid fracture	Radial plating and DRUJ pinning	Open reduction	Free bone debrided and suture anchor fixation
Shiboi et al. [10]	2005	34/M	Fall from 2 m height	Apex volar type	Posteromedial dislocation	–	Radial plating	Closed reduction	–
Sarup and Bryant [6]	1997	35/F	Fall from flight of stairs	unknow	Posterolateral dislocation	Humeral shaft and ulnar styloid fracture	Radial plating	Closed reduction	Humeral intramedullary fixation

**Fig. 5:**
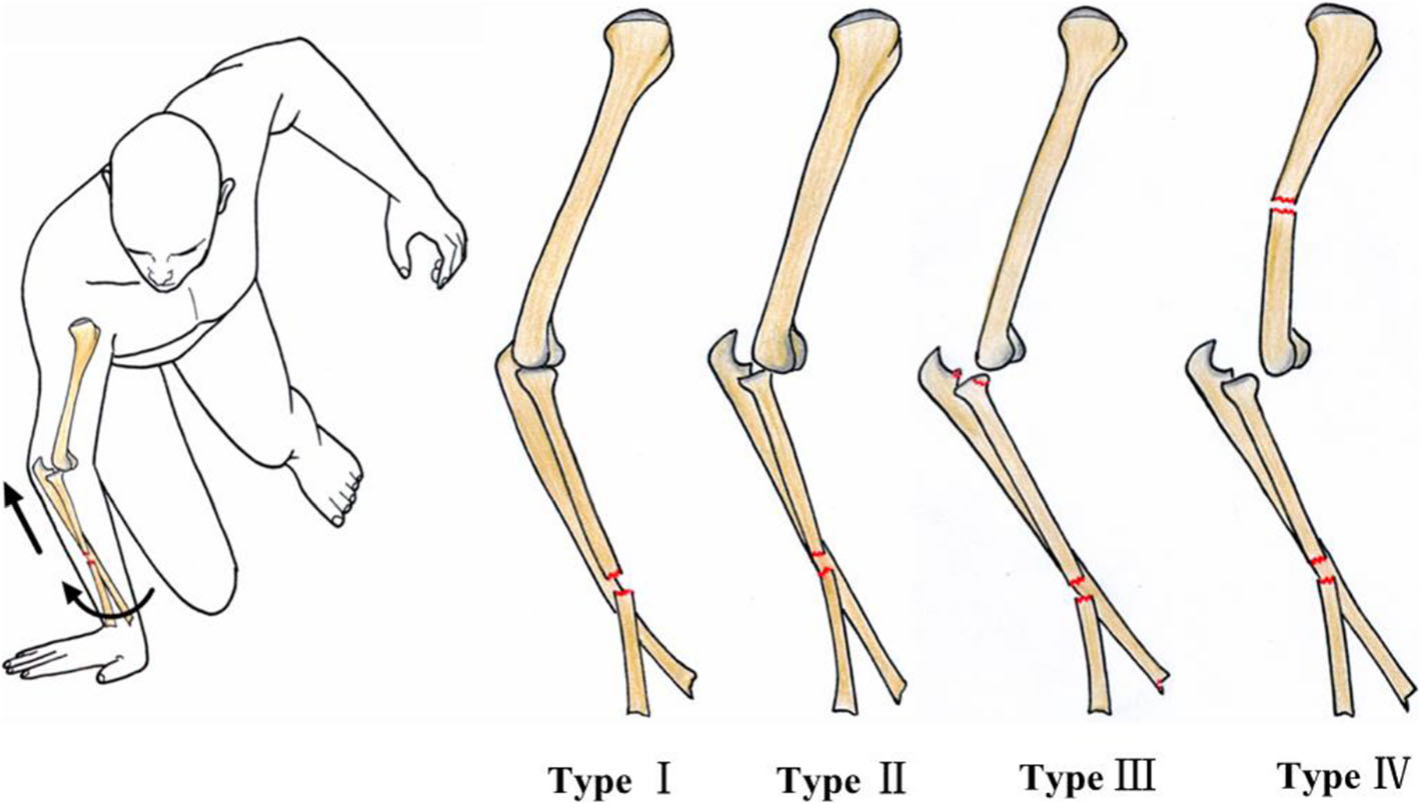
The “floating ulna” injure classified into four types, take apex-palmar type Galeazzi fracture for example

According to the literatures and the levers from forceful transmission of axial force starting from the wrist and extending to the elbow, a classification of four types of “floating ulna” injuries was recommended: Type I: Ipsilateral Galeazzi fracture and instability of the elbow; Type II: Ipsilateral Galeazzi fracture and elbow dislocation; Type III: Ipsilateral Galeazzi fracture, elbow dislocation, and forearm fractures, in which the forearm fracture include only those fractures around the elbow (radial head and coronoid fracture) or around the wrist (scaphoid and ulnar styloid fracture), and the shaft fracture of the ulna and proximal and medial fracture of the radius are excluded; Type IV: Ipsilateral Galeazzi fracture, elbow dislocation, and fracture of the humerus (Fig. 5).

This case of “floating ulna” injury was likely caused by significant amount of deforming force and the unique position of upper limb when the patient fell from a height of 1–2 m in high-energy trauma.

## Data Availability

Data sharing not applicable to this article as no datasets were generated or analysed during the current study.

## Abbreviations

DRUJ: The distal radioulnar joint
CT: Computerized tomography
K-wire: Kirchner wire
